# Postconditioning inhibits myocardial apoptosis during prolonged reperfusion via a JAK2-STAT3-Bcl-2 pathway

**DOI:** 10.1186/1423-0127-18-53

**Published:** 2011-08-02

**Authors:** Yikui Tian, Wenjuan Zhang, Dachuan Xia, Paul Modi, Degang Liang, Minxin Wei

**Affiliations:** 1 Department of Cardiovascular Surgery, Tianjin Medical University General Hospital, Tianjin, P. R. China; 2 Department of Cardiology, Tianjin Medical University General Hospital, Tianjin, P. R. China; 3 Department of Cardiac Surgery, Liverpool Heart and Chest Hospital, Liverpool, UK

**Keywords:** Ischemia/reperfusion injury, apoptosis, postconditioning, JAK2-STAT3 pathway, Bcl-2

## Abstract

**Background:**

Postconditioning (PostC) inhibits myocardial apoptosis after ischemia-reperfusion (I/R) injury. The JAK2-STAT3 pathway has anti-apoptotic effects and plays an essential role in the late protection of preconditioning. Our aim was to investigate the anti-apoptotic effect of PostC after prolonged reperfusion and the role of the JAK2-STAT3 pathway in the anti-apoptotic effect of PostC.

**Methods:**

Wistar rats were subjected to 30 minutes ischemia and 2 or 24 hours (h) reperfusion, with or without PostC (three cycles of 10 seconds reperfusion and 10 seconds reocclusion at the onset of reperfusion). Separate groups of rats were treated with a JAK2 inhibitor (AG490) or a PI3K inhibitor (wortmannin) 5 minutes before PostC. Immunohistochemistry was used to analyze Bcl-2 protein levels after reperfusion. mRNA levels of Bcl-2 were detected by qRT-PCR. TTC staining was used to detect myocardial infarction size. Myocardial apoptosis was evaluated by TUNEL staining. Western-blot was used to detect p-STAT3 and p-Akt levels after reperfusion.

**Results:**

There was more myocardial apoptosis at 24 h *vs *2 h after reperfusion in all groups. PostC significantly reduced myocardial apoptosis and elevated Bcl-2 levels at both 2 and 24 hours after reperfusion. PostC increased p-STAT3 and p-Akt levels after reperfusion. Administration of AG490 reduced p-STAT3 and p-Akt levels and attenuated the anti-apoptotic effect of PostC. Wortmannin also reduced p-Akt levels and attenuated the anti-apoptotic effect of PostC but had no effect on p-STAT3 levels. AG490 abrogated the up-regulation of Bcl-2 by PostC.

**Conclusion:**

PostC may reduce myocardial apoptosis during prolonged reperfusion via a JAK2-STAT3-Bcl-2 pathway. As a downstream target of JAK2 signaling, activation of PI3K/Akt pathway may be necessary in the protection of PostC.

## Background

Postconditioning (PostC), defined as transient periods of ischemia and reperfusion at the onset of reperfusion, has been shown to be protective against myocardial ischemia-reperfusion (I/R) injury in multiple species [1]. Recent studies reported that the cardioprotective effects of PostC persisted after prolonged reperfusion [2,3]. Cardiomyocyte apoptosis is one of the major mechanisms underlying I/R injury. The progressive loss of cardiomyocytes due to apoptosis plays a critical role in cardiac dysfunction after acute myocardial infarction. Previous studies have reported that PostC inhibits apoptosis in both in vivo and in vitro models [4,5]. However, the anti-apoptotic effect of PostC after prolonged reperfusion has not yet been well defined.

The Janus kinase (JAK)-signal transducers and activators of transcription (STAT) pathway is an evolutionary conserved signaling network involved in a wide range of distinct cellular processes, including inflammation, apoptosis, cell-cycle control and development. As a part of SAFE (Survivor Activating Factor Enhancement) pathway, the JAK2-STAT3 pathway has anti-apoptotic effects and plays essential roles in postconditioning and the late protection of preconditioning [6-8]. However, the role of the JAK2-STAT3 pathway in the anti-apoptotic effects of PostC is not yet fully understood. The present study was designed to investigate the anti-apoptotic effect of PostC after prolonged reperfusion and to define the role of the JAK2-STAT3 pathway in this.

## Methods

All animals were obtained from the Chinese People's Liberation Army Academy of Military Medical Sciences. The experimental protocol was approved by the Tianjin Medical University Animal Care and Use Committee. Male Wistar rats weighing 240 to 280 g were anesthetized with sodium pentobarbital (40-50 mg/kg, intraperitoneal) and ventilated with oxygen-enriched room air using a rodent ventilator. The left carotid artery was cannulated for monitoring arterial pressure and electrocardiogram (ECG) leads were placed to record heart rate. The chest was opened by a left thoracotomy in the fifth intercostal space. After pericardiotomy, a 6-0 prolene ligature was placed under the left coronary artery (LCA) where it emerges from beneath the left atrial appendage and the ends were threaded through a small plastic tube to form a snare for reversible LCA occlusion. Complete LCA occlusion was confirmed by observing cyanosis of the myocardium as well as ST-segment deviation.

### Experimental protocol

**Figure 1:**
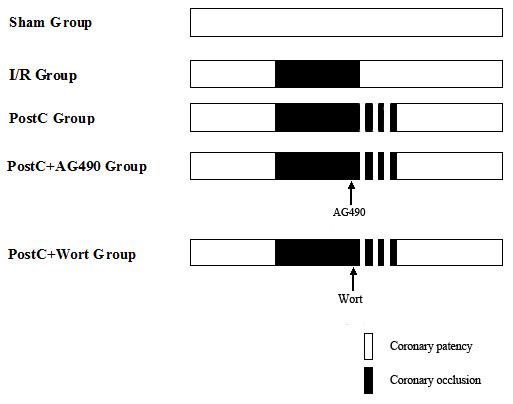
**Experimental groups and their protocols**. Rats were randomly divided into five groups as showed in the figure.

Rats were randomized into five groups, as shown in Figure 1:

(1) sham group - the ligature was placed under the LCA without occlusion;

(2) I/R group - no interventions were applied either before or after LCA occlusion;

(3) PostC group - three cycles of 10 s of reperfusion and 10 s of reocclusion immediately at the onset of reperfusion;

(4) PostC+Ag490 group - the JAK2 inhibitor AG 490 (Sigma-Aldrich, St. Louis, MO, dissolved in 0.1% DMSO solution, 3 mg/Kg, iv) was administered 5 min before PostC;

(5) PostC+wortmannin group - the PI3K inhibitor wortmannin (Sigma-Aldrich, St. Louis, MO, dissolved in 0.1% DMSO solution, 1.5 mg/Kg, ip) was administered 5 min before PostC.

Other two control groups, treating rats with Ag490 or wortmannin alone without PostC, were designed to evaluate the effects of Ag490 and wortmannin alone in I/R group. The doses of AG490 (3 mg/Kg,iv) and wortmannin (1.5 mg/Kg, ip) were according to previous study [9]. Additional rats from the sham and PostC groups were treated with 0.1% DMSO to detect any independent effects of the DMSO vehicle. Myocardial biopsies from the sham group were obtained at 20 min, 2.5 h or 24 h after thoracotomy (n = 4 at each time point). The other groups were subjected to 30 min of LCA occlusion followed by 10 min (n = 8, for signaling pathway detections), 2 h (n = 8, for TUNEL and Bcl-2 detections) or 24 h (n = 8, for TUNEL and Bcl-2 detections) of reperfusion. Myocardial biopsies were taken at the end of the experiment. The PostC protocol was performed with three cycles of 10 s of reperfusion and 10 s of reocclusion immediately at the onset of reperfusion as reported previously [4].

### Myocardial infarction size analysis

After reperfusion, the LCA was re-ligated at its original site. 2 ml of 2% evans blue was injected into the inferior vena cava to define the area at risk (AAR). The ventricles of the hearts were sliced transversely into 2-mm thick slices. The slices were incubated in 1% triphenyltetrazolium chloride (TTC, sigma) at 37°C for 15 min to identify the infarction size (IS). AAR and IS were determined by computerized planimetry using ImageJ software. AAR was expressed as a percentage of the left ventricle and IS was expressed as a percentage of the AAR.

### TUNEL (Terminal Doxynucleotidyl Transferase-mediated dUTP-X Nick End Labeling) staining

The histochemical detection of apoptotic cells was performed as previously reported [4]. The tissue blocks were fixed in 4% paraformaldehyde and incubated with proteinase K. Fragments of DNA in the tissue sections were analyzed using a TUNEL detection kit (Promega Corporation, Madison, WI). For each slide, the color images of 10 separate fields were captured randomly and digitized. The cells with clear nuclear labeling were defined as TUNEL-positive cells. The apoptotic index was calculated as the number of TUNEL-positive cells/total number of myocytes × 100.

### Immunohistochemistry

Transmural LV samples were embedded in paraffin and cut into 5μm sections. Detection of Bcl-2 expression was performed as described previously [10]. Tissue sections were exposed overnight to rabbit polyclonal anti-bcl-2 antibody (Santa Cruz Biotechnology, Santa Cruz, CA) at 4°C, washed in PBS and then incubated with biotinylated goat antirabbit IgG for 60 min at 37°C. After two washing steps, sections were exposed to streptavidin-horseradish-peroxidase complex for 30 min at 37°C, and then visualized with 3,3'-diaminobenzidine, embedded in glycerol gelatin and coverslips applied. Images were captured digitally and analyzed using Image-Pro Plus version 6.0 (Media Cybernetics, Inc, Bethesda, MD). The result was expressed as the ratio of positive to negative staining area.

### Western-blot analysis

Transmural LV samples were obtained after 10 min of reperfusion. Western blotting was performed as described previously (5). In brief, freshly frozen myocardial tissue samples were homogenized in RIPA buffer. Total protein was separated by 10% SDS-PAGE and transferred to nitrocellulose membranes. Membranes were exposed to p-Akt, Akt, p-STAT3(Tyr705) or STAT3 antibody(Cell Signaling Technology, Beverly MA), and subsequently incubated with a chemiluminescence substrate and exposed to radiographic film. The images were captured digitally and the density at specific molecular weights was measured using Gel-Pro analyzer version 3.0 (Media Cybernetics, Inc, Bethesda, MD).

### Quantitative reverse transcriptase-polymerase chain reaction

Bcl-2 mRNA levels were determined by quantitative reverse transcriptase-polymerase chain reaction (qRT-PCR). For extraction of total RNA the RNeasy Mini Kit (QIAGEN China Co., Ltd, Shanghai) was used according to the manufacturer's instructions. cDNAs were synthesized using RevertAid™ First Strand cDNA Synthesis Kit(Fermentas China Co., Ltd. Shenzhen). qPCR was performed with Maxima^® ^SYBR Green qPCR Master Mix, ROX Solution provided(Fermentas China Co., Ltd. Shenzhen). Fluorescent signals were normalized to an internal reference, and the threshold cycle (Ct) was set within the exponential phase of the PCR. The relative gene expression was calculated by comparing cycle times for each target PCR. The target PCR Ct values were normalized by subtracting the GAPDH Ct value, which provided the ΔCt value. mRNA levels were quantified with the 2^(-ΔΔct) ^relative quantification method. The following primers were used for detection: Bcl-2 FW: CCGGGAGATCGTGATGAAGT, RV: ATCCCAGCCTCCGTTATCCT. GAPDH FW: ACTTTGTCAAGCTCATTTCCTG, RV: CTCTCTTCCTCTTGTGCTCTTG.

### Statistical analysis

All data are expressed as mean ± SEM and analyzed using SPSS 15.0 (Chicago, IL). Independent samples *t*-test and one-way ANOVA were used to compare data with post hoc analysis using the Student-Newman-Keuls correction. A p-value <0.05 was considered significant.

## Results

### Hemodynamic data (Table 1)

There were no baseline differences. After 30 min ischemia, mean arterial blood pressure decreased significantly between the sham and intervention groups. No statistical differences were observed among all groups at both 2 and 24 hours after reperfusion.

**Table 1:** Hemodynamic Data

Measurements	Baseline		Ischemia		2 h after reperfusion		24 h after reperfusion	
	
Groups	*HR (Beat/min)*	*MABP (mmHg)*	*HR (Beat/min)*	*MABP (mmHg)*	*HR (Beat/min)*	*MABP (mmHg)*	*HR (Beat/min)*	*MABP (mmHg)*
Sham-10 m	381 ± 5	98 ± 3	382 ± 4	97 ± 3	-		-	-

Sham-2 h	373 ± 5	98 ± 4	380 ± 5	96 ± 3	379 ± 4	96 ± 2	-	-

Sham-24 h	373 ± 3	97 ± 4	373 ± 3	96 ± 4	371 ± 3	97 ± 3	368 ± 4	95 ± 3

Control-10 m	376 ± 4	97 ± 4	382 ± 4	81 ± 2*	-	-	-	-

Control-2 h	373 ± 5	96 ± 3	379 ± 5	82 ± 3*	371 ± 5	96 ± 3	-	-

Control-24 h	374 ± 5	99 ± 2	379 ± 5	81 ± 2*	378 ± 3	98 ± 3	373 ± 2	96 ± 3

Post-10 m	374 ± 4	96 ± 4	383 ± 4	82 ± 3*	-	-	-	-

Post-2 h	379 ± 4	95 ± 5	389 ± 4	81 ± 3*	381 ± 4	96 ± 4	-	-

Post-24 h	375 ± 4	96 ± 4	383 ± 4	82 ± 2*	374 ± 4	96 ± 4	375 ± 3	94 ± 3

Post+Ag490-10 m	374 ± 5	97 ± 3	381 ± 4	81 ± 3*	-	-	-	-

Post+Ag490-2 h	381 ± 5	98 ± 2	383 ± 6	80 ± 2*	377 ± 5	96 ± 2	-	-

Post+Ag490-24 h	379 ± 4	98 ± 2	382 ± 4	86 ± 3*	376 ± 5	93 ± 2	376 ± 4	95 ± 3

Post+wort-10 m	376 ± 6	97 ± 3	380 ± 6	79 ± 3*	-	-	-	-

Post+wort-2 h	377 ± 5	97 ± 3	386 ± 4	80 ± 3*	375 ± 4	96 ± 3	-	-

Post+wort-24 h	373 ± 6	97 ± 2	380 ± 6	86 ± 2*	368 ± 5	97 ± 3	369 ± 3	95 ± 3

*p < 0.05 compared with baseline. HR = heart rate, MABP = mean artery blood pressure.

### Myocardial infarction after prolonged reperfusion

**Figure 2:**
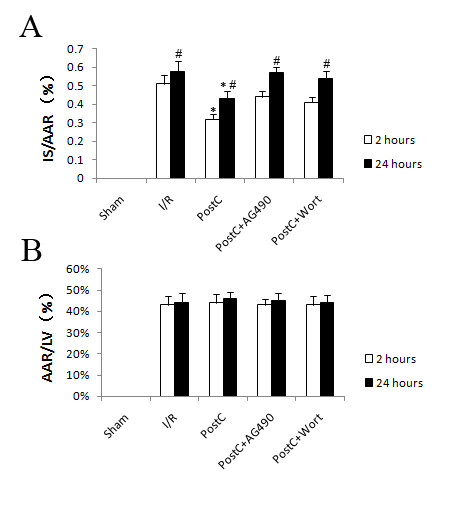
**Myocardial infarction size at 2 and 24 hours after reperfusion (n = 8 at each time point)**. Myocardial infarction size was detected using TTC staining. Myocardial infarction area was increased between 2 and 24 hours after reperfusion in all intervention groups. PostC significantly reduced myocardial infarction size after reperfusion. Both AG490 (a selective JAK2 inhibitor) and Wortmannin (a selective PI3K inhibitor) abolished cardioprotection of PostC. *p < 0.05 compared with I/R-2 h and I/R-24 h groups respectively; #p < 0.05 compared with I/R-2 h, PostC-2 h, PostC+AG490-2 h and PostC+wort-2 h groups respectively. IS = Infarction size, LV = Left ventricle, AAR = Area at risk.

No myocardial infarction was found in sham group. Area of risk was similar among all treatment groups. Myocardial infarction area was increased between 2 and 24 hours after reperfusion in all intervention groups. PostC significantly reduced myocardial infarction size after reperfusion (Figure 2). Treatment with AG490 and wortmannin alone had no effect on myocardial infarction after reperfusion in I/R group.

### Myocardial apoptosis after prolonged reperfusion

**Figure 3:**
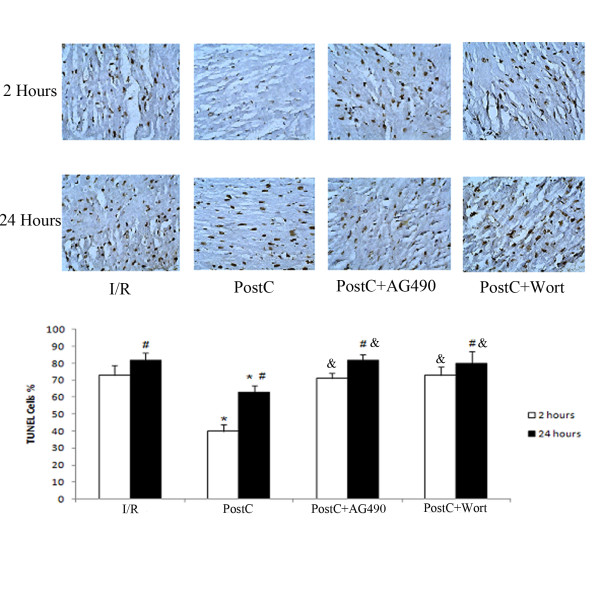
**Myocardial apoptosis at 2 and 24 hours after reperfusion (n = 8 at each time point)**. Myocardial apoptosis was detected by TUNEL staining. TUNEL-positive cells (black staining) increased during prolonged reperfusion. PostC significantly reduced myocardial apoptosis at both 2 and 24 hours after reperfusion. AG490 and Wortmannin attenuated the anti-apoptotic effects of PostC. *p < 0.05 compared with I/R-2 h and I/R-24 h groups respectively; #p < 0.05 compared with I/R-2 h, PostC-2 h, PostC+AG490-2 h and PostC+wort-2 h groups respectively; &p < 0.05 compared with PostC-2 h and PostC-24 h respectively.

There were no TUNEL-positive cells in the sham group. There was more apoptosis at 24 h compared to 2 h reperfusion in all intervention groups (Figure 3). PostC significantly reduced myocardial apoptosis at 2 and 24 h after reperfusion. Treating rats with AG490 or wortmannin significantly attenuated anti-apoptosis effects of PostC. However, AG490 or wortmannin alone had no effect on apoptosis index after reperfusion in I/R group.

### Myocardial Bcl-2 levels

**Figure 4:**
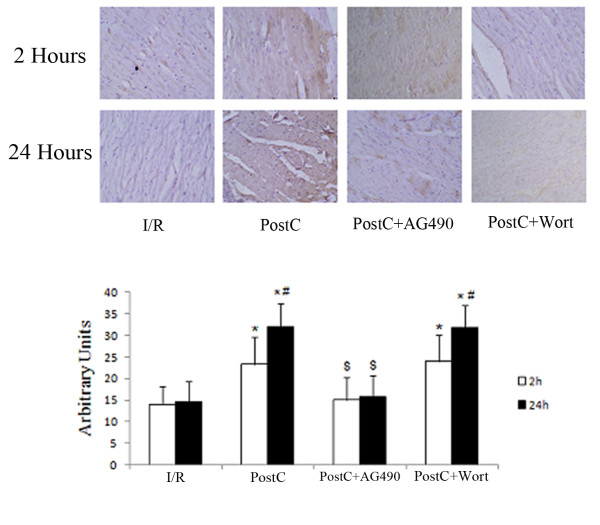
**Protein levels myocardial Bcl-2 at 2 and 24 hours after reperfusion (n = 8 at each time point)**. Bcl-2 levels were detected by immunohistochemical staining. PostC elevated Bcl-2 expression in myocardial (brown staining) at both 2 and 24 hours after reperfusion. AG490 abrogated the up-regulation of Bcl-2 levels by PostC. Wortmannin had no effects on Bcl-2 levels. *p < 0.05 compared with I/R-2 h and I/R-24 h groups respectively; #p < 0.05 compared with PostC-2 h and PostC+wort-2 h groups respectively; $p < 0.05 compared with PostC-2 h and PostC-24 h groups respectively.

**Figure 5:**
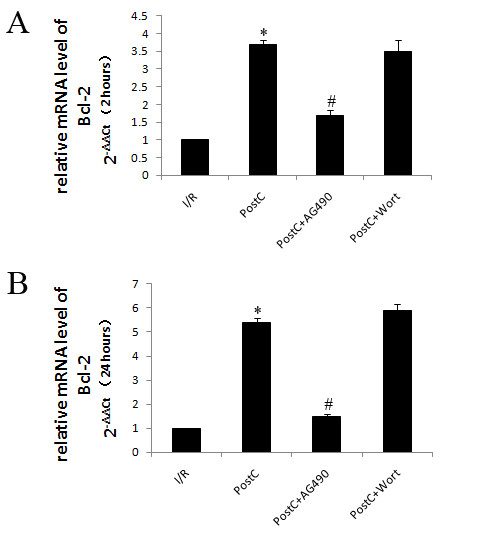
**mRNA levels of Bcl-2 at 2 and 24 hours after reperfusion (n = 8 at each group)**. mRNA levels of Bcl-2 were analysis by qRT-PCR. PostC increased Bcl-2 level after reperfusion. AG490 abrogated the up-regulation of Bcl-2 levels by PostC. Wortmannin had no effects on Bcl-2 levels. *p < 0.05 compared with I/R-2 h and I/R-24 h groups respectively. #p < 0.05 compared with PostC groups.

Both protein and mRNA levels of Bcl-2 were similar at 2 and 24 h in the control group. PostC elevated levels of Bcl-2 after reperfusion, which increased between 2 and 24 h after reperfusion. The JAK2 inhibitor AG490 decreased Bcl-2 levels after reperfusion. The PI3K inhibitor wortmannin had no effects on Bcl-2 levels (Figure 4, 5).

### Correlation between JAK2-STAT3 and PI3K/Akt signaling pathways in PostC

**Figure 6:**
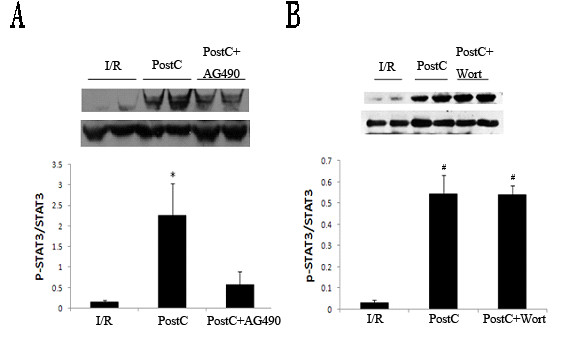
**Levels of p-STAT3 at 10 minutes after reperfusion (n = 8 each group)**. p-STAT3 levels were analyzed by western blot. PostC activated JAK2-STAT3 pathway. (A), JAK2 inhibitor AG490 reduced p-STAT3 levels after PostC. (B), PI3K inhibitor wortmannin had no effects on p-STAT3 levels. *p < 0.05 compared with I/R and PostC+AG490 groups. #p < 0.05 compared with I/R group.

**Figure 7:**
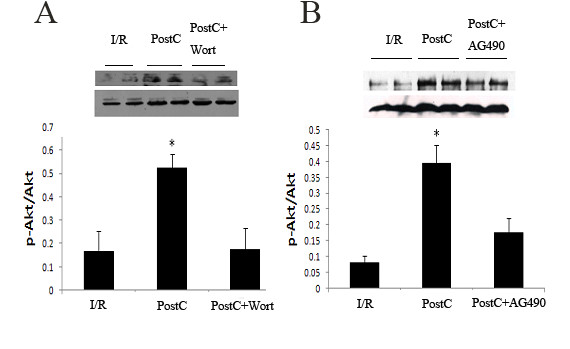
**Levels of p-Akt at 10 minutes after reperfusion (n = 8 each group)**. p-Akt levels were analyzed by western blot. PostC activated PI3K/Akt pathway. (A), PI3K inhibitor wortmannin reduced p-Akt levels after PostC. (B), JAK2 inhibitor AG490 reduced p-Akt levels after PostC. *p < 0.05 compared with I/R and PostC+Drug groups respectively.

PostC significant increased the expression of p-STAT3 and p-Akt. Administration of AG490 before PostC reduced p-STAT3 and p-Akt levels and attenuated the cardioprotection effect of PostC. Wortmannin also reduced p-Akt levels and attenuated the cardioprotection effect of PostC but had no effect on p-STAT3 levels (Figures 6 and 7).

## Discussion

The present study demonstrated that apoptosis following I/R injury increases after prolonged reperfusion. The prolonged anti-apoptotic effect of PostC may be related to elevation of Bcl-2 24 h after reperfusion which is regulated by the JAK2-STAT3 pathway. PI3K/Akt pathway, regulated by JAK2 signaling, may be necessary in the protection of PostC.

Previous studies have shown that oxidative stress, Ca^2+ ^overload, pH paradox and inflammation during early reperfusion are the major mediators of lethal injury which indicates that this period is important in the pathogenesis of reperfusion injury [11]. Zhao *et al*. recently found that infarction size increases significantly between 6 h and 24 h after reperfusion in a canine model. In agreement with this, the present study also found that myocardial apoptosis increases after prolonged reperfusion in rat hearts. Myocardial reperfusion injury may increase with the duration of reperfusion. However, Argaud *et al*. found that myocardial infarction size had no difference between 4 h and 72 h after reperfusion in rabbit hearts. The difference in animal species and the procedures to induce ischemia/reperfusion injury are considered to be the reasons for these different results [2,12,13].

PostC was first described by Johansen's group in 2003 [14]. After that, the ability of PostC to protect myocardium from I/R injury has been confirmed by several studies and in multiple species. PostC not only reduces infarction size but also limits apoptosis after reperfusion. Recent studies reported that PostC affords persistent infarction size reduction after prolonged reperfusion in both canine models and humans [2,3]. But they did not distinguish whether this long-term cardioprotection was a continued effect of the early phase or an independent effect. The present results suggested that PostC could limit myocardial apoptosis after reperfusion. Bcl-2 levels were significantly up-regulated by PostC. Interestingly, there was an increase in Bcl-2 levels between 2 and 24 hours after reperfusion in PostC groups. This indicated that PostC may reduce myocardial apoptosis during prolonged reperfusion via up-regulated anti-apoptotic factors such as Bcl-2. Further study focus on other apoptosis-associated proteins such as Bcl-x, BAD and BAX may be helpful to define the long-term benefit of PostC.

The anti-apoptotic effects of the JAK2-STAT3 signaling pathway have been shown by several studies in tumors. Several apoptosis-related protein genes, such as Bcl-2 and Bcl-xl, have been identified as target genes of STAT3 [15,16]. Many studies have demonstrated that ischemic preconditioning upregulates COX-2 and iNOS at 24 hours after intervention, which is dependent on transcriptional regulation by the JAK-STAT pathway [17,18]. The present results showed that PostC significantly activated STAT3 after reperfusion and AG490 attenuated the anti-apoptotic effect of PostC. Elevation of Bcl-2 after reperfusion required STAT3 activation which indicated that PostC may afford a persistent anti-apoptotic effect via a JAK2-STAT3-Bcl-2 pathway.

Activation of the PI3K/Akt pathway prevents cardiac myocyte apoptosis and protects the myocardium from I/R injury. Furthermore, as the major component of the RISK (Reperfusion Injury Salvage Kinase) pathway, the PI3K/Akt pathway has been shown to play a crucial role in PostC [19-21]. Goodman *et al*. demonstrated that JAK-STAT signaling may provide upstream initiation of RISK pathway signaling via PI3K-Akt activation, and JAK-STAT signaling is insufficient on its own to provide cardioprotection following PostC without subsequent RISK activation [7]. In accordance with this, the present study showed that JAK2 signaling regulated the activation of the PI3K/Akt pathway in PostC. Blocking the PI3K/Akt pathway attenuated the cardioprotection of PostC at all time points. However, wortmannin had no effect on Bcl-2 levels after PostC. The possible explanation of these results is that activation of JAK2-STAT3-Bcl-2 pathway alone may be not sufficient to limit apoptosis without activation of PI3K/Akt pathway.

## Conclusions

In conclusion, the present study demonstrated that PostC may reduce myocardial apoptosis during prolonged reperfusion via a JAK2-STAT3-Bcl-2 pathway. As a downstream target of JAK2 signaling, the activation of PI3K/Akt pathway may be necessary in the protection of PostC. Time-course experiments in further study may be helpful to demonstrate precise roles of the pathways in prolonged protection of PostC. However, our results indicated that the protective effect of PostC is not enough to prevent myocardial apoptosis during prolonged reperfusion. Combination therapy targeting apoptosis during prolonged reperfusion may provide more effective protection.

## Competing interests

The author declares that they have no competing interests.

## Authors' contributions

YT participated in the design of the study and drafted the manuscript, carried out the surgery of animals. WZ carried out the TTC staining, TUNEL staining and Immunohistochemistry, participated in the sequence alignment. DX carried out the Western-blot and qRT-PCR. PM did the language check of the manuscript. DL performed the statistical analysis. MW conceived of the study, and participated in its design and coordination. All authors read and approved the final manuscript.
